# Improvement in Quality of Life Using an Ethylene–Vinyl Acetate Palatal Obturator in a Patient with Self-Inflicted Nasal Defect and Palatal Perforation

**DOI:** 10.4317/jced.64151

**Published:** 2026-07-29

**Authors:** Naohiro Horie, Kaoru Kusano, Kenji Yokozeki, Kazumi Osada, Hiroki Nagayasu, Tsuyoshi Shimo, Masaru Murata

**Affiliations:** 1 Division of Reconstructive Surgery for Oral and Maxillofacial Region, School of Dentistry, Health Sciences University of Hokkaido; 2 Department of Oral Implantology, Osaka Dental University; 3 Division of Occlusion and Removable Prosthodontics, Department of Oral Rehabilitation, School of Dentistry, Health Sciences University of Hokkaido; 4 Laboratory of Food Science & Nutrition, Department of Food Biosciences and Biotechnology, College of Bioresource Sciences. Nihon University; 5 Division of Oral and Maxillofacial Surgery, Department of Human Biology and Pathophysiology, School of Dentistry, Health Sciences University of Hokkaido; 6 Division of Oral Regenerative Medicine, Department of Human Biology and Pathophysiology, School of Dentistry, Health Sciences University of Hokkaido

## Abstract

A 67-year-old female patient with intellectual disability presented with a self-inflicted nasal defect and palatal perforation. In 2006, she was referred to our department by a nurse from her care facility with the chief complaint of food leakage into the nasal cavity due to the palatal perforation. Her medical history revealed a long-standing habit of scratching the nasal cavity, which had persisted for more than 30 years since approximately 27 years of age. Clinical and radiographic examinations, including three-dimensional computed tomography (3D-CT), demonstrated a nasal defect, crust formation around the nasal region, a perforation in the central palate (approximately 3 × 4 mm), and a palatal bone defect (approximately 28 × 24 mm). We selected a conservative approach using a soft palatal obturator made of ethylene-vinyl acetate (thickness: 3.0 mm) instead of surgical closure. In 2007, after several sessions of assisted feeding training with verbal encouragement, the patient accepted the use of the obturator. Although enlargement of the perforation (approximately 12 × 9 mm) was observed in 2018, her body weight increased by 4.0 kg and body mass index (BMI) improved by 3.1 compared with baseline values in 2006. In addition, no hospitalization due to aspiration pneumonia or other systemic complications occurred during the approximately 18-year follow-up period. The patient died at the age of 84 in 2024. The long-term use of a palatal obturator in a care facility setting may have contributed to improved nutritional status and reduced self-injurious behavior.

## Introduction

Self-injurious behavior is defined as the intentional act of causing harm to one's own body and differs from suicide in that it is generally non-lethal. Examples include wrist cutting, nail removal, burning the skin with a lighter, and hair pulling. Psychiatric disorders and intellectual disability are known to be associated with self-injurious behavior, and in the head and neck region, self-inflicted injuries to the lips and tongue have been frequently reported (1). However, severe cases involving both nasal defects and palatal perforation are very rare. The management of self-inflicted palatal perforation is clinically a challenging case, because her persistent self-injurious behavior may lead to recurrence even after a surgical closure. Therefore, we believe a long-term functional rehabilitation using a conservative approach may be more appropriate in selected patients. In this report, we describe a patient presenting both a self-inflicted nasal defect and palatal perforation with intellectual disability. Rather than the surgical closure, we selected the conservative management using a palatal obturator for a long-term functional rehabilitation. Since 2007, the patient has accepted the use of an ethylene-vinyl acetate palatal obturator and has been able to consume meals independently at her care facility. During regular follow-up visits at the Dental Clinic of Health Sciences University of Hokkaido, effective communication was achieved among the patient, attending nurse, and clinicians through the use of the obturator. The patient maintained an adequate quality of dietary life at the care facility for approximately 18 years until her death, and this report describes the clinical course and the long-term functional outcome of the conservative obturator management.

## Case Report

**Figure 1:**
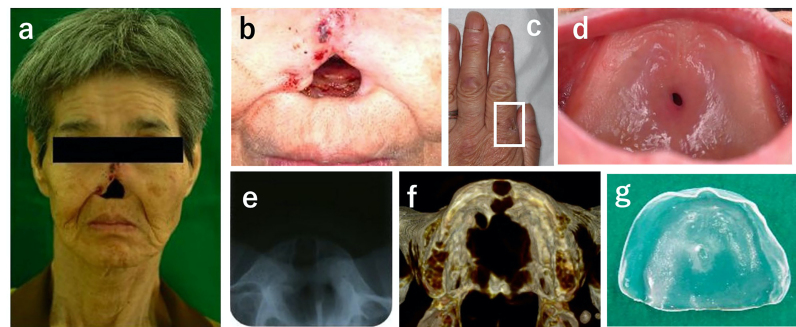
Clinical findings at the initial presentation and the fabricated intraoral appliance (2006 at age 67). (a) Facial photograph; (b) close-up view of the nasal defect; (c) crusted lesion on the dorsal aspect of the left hand (white box); (d) maxillary intraoral photograph; (e) maxillary occlusal radiograph; (f) three-dimensional computed tomography (3D-CT) image; (g) palatal obturator.

A 67-year-old female patient presented to our department in August 2006 with the chief complaint of a "hole in the upper jaw," as reported by staff at her care facility. Her family history was unremarkable. Her medical history included meningitis at the age of 3 years and intellectual disability. According to the clinical history, due to her intellectual disability, the patient had engaged in self-injurious behavior since approximately 27 years of age, repeatedly scratching the nasal region with her fingers. This behavior, particularly involving scratching within the nasal cavity, had persisted for more than 30 years, leading to gradual necrosis from the base of the nasal ala. At approximately 57 years of age, complete loss of the nasal structure occurred, and she was evaluated at a nearby otorhinolaryngology department, where no neoplastic or other pathological abnormalities were identified. In August 2006, care facility staff noticed food entering the nasal cavity after meals, extending from the nasal floor to the inferior turbinate, and the patient was referred to our dental clinic for further evaluation and treatment. - Clinical findings General examination revealed a height of 148.4 cm, body weight of 31.5 kg, and a body mass index (BMI) of 14.0, indicating an underweight condition. Extraoral examination showed a nasal defect and crust formation on the surrounding nasal skin. Redness of the mucosa corresponding to the left inferior turbinate was observed. Crust formation was also noted on the fingers (Fig. 1a-c).

1

**Figure 2:**
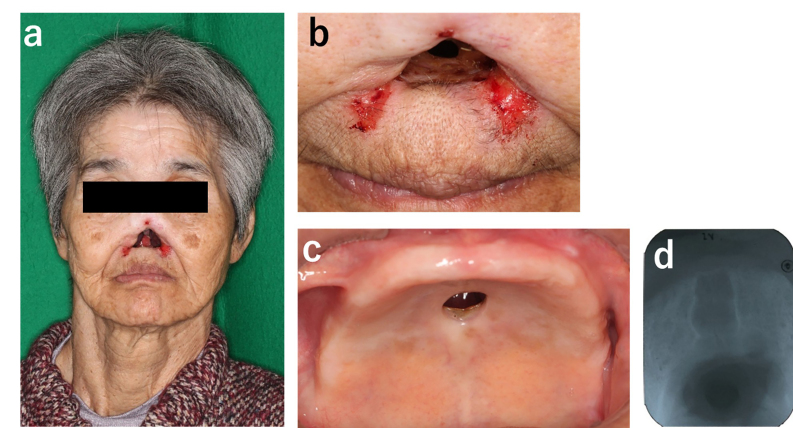
Clinical findings at the 9-year follow-up (2015 at age 76). (a) Facial photograph; (b) close-up view of the nasal defect; (c) maxillary intraoral photograph; (d) maxillary occlusal radiograph.

**Figure 3:**
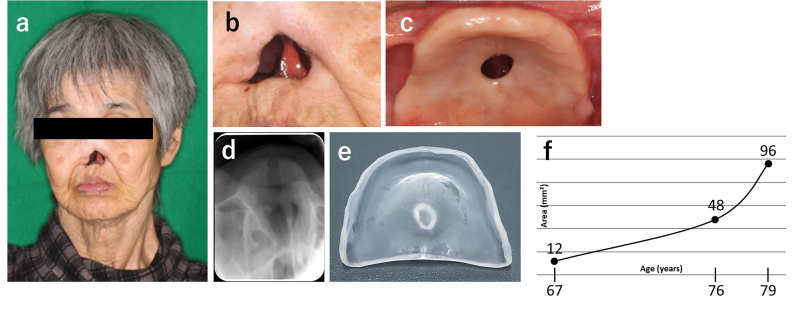
Clinical findings at the 12-year follow-up and the intraoral appliance currently in use (2018 at age 79). (a) Facial photograph; (b) close-up view of the nasal defect; (c) maxillary intraoral photograph; (d) maxillary occlusal radiograph; (e) palatal obturator; (f) Size of the palatal perforation (y-axis: area; x-axis: age).

Intraoral examination revealed a tissue defect (perforation) in the central palate measuring approximately 3 × 4 mm, communicating with the nasal cavity (Fig. 1d). Both the maxilla and mandible were edentulous, with no evidence of erosion or ulceration of the palatal mucosa. The patient had no prior experience with dentures. She was able to communicate verbally and did not report pain. Radiographic examination showed a radiolucent area suggestive of a bone defect at the midline palatal suture on occlusal radiographs, without continuity with the incisive foramen (Fig. 1e). Three-dimensional computed tomography (3D-CT) revealed a palatal bone defect in the midline (approximately 28 × 24 mm) and a defect of the nasal septum (Fig. 1f). Laboratory tests were negative for syphilis, and microbiological culture showed no evidence of fungal infection. The clinical diagnosis was a nasal defect with palatal perforation caused by self-injurious behavior in a patient with intellectual disability. - Treatment and clinical course In April 2007, after obtaining an impression of the maxilla, a soft palatal obturator made of ethylene-vinyl acetate (thickness: 3.0 mm) was fabricated in our dental laboratory (Fig. 1g). In May 2007, the obturator was first fitted in the outpatient clinic, demonstrating good adaptation. Although the patient initially showed resistance to wearing the obturator, she gradually accepted it after sufficient communication and several training sessions. Feeding training using her favorite food (banana) was conducted, and it was confirmed, together with the accompanying nurse, that food no longer entered the nasal cavity while wearing the obturator. Instructions on the use and maintenance of the obturator were subsequently provided to both the patient and the care staff. At the follow-up visit one month later, the obturator had changed color from transparent to yellow. Based on dietary history, this discoloration was attributed to curry consumption. The patient was followed monthly until 2007 and subsequently every three months from 2008. At each visit, the perforation site, presence of ulceration, and fit of the obturator were evaluated. The obturator was replaced every three months, and its use prevented food leakage into the nasal cavity and resulted in mild improvement in hypernasality. Around 2014, pharmacological cognitive behavioral therapy was attempted at a local psychiatric clinic; however, no significant effect was observed. According to care staff, self-injurious behavior persisted when the patient was unattended, and in recent years, it occasionally involved the toenails. The size of the palatal perforation increased from 12 mm² at the initial visit to 48 mm² after 9 years (2015) and 96 mm² after 12 years (2018). Despite this enlargement, self-injurious behavior around the nasal defect gradually decreased, and no crust formation was observed in this region after 12 years (Figs. 2a,b, 3a,b,f).

2

3

The patient's body weight increased to 35.8 kg and BMI to 17.1, indicating improved nutritional status. She remained in good general health without hospitalization for pneumonia or other conditions for approximately 18 years and continued to accept the use of the obturator. The patient died of senescence in 2024.

## Discussion

The nasal defect in the present patient was not attributable to congenital factors or pathological disease, but rather resulted from acquired self-injurious behavior. At the initial visit, information obtained from the accompanying care staff indicated that the patient had resided in a care facility for approximately 40 years due to intellectual disability and had a history of self-injurious behaviors, including nail removal. Although surgical closure of the oro-nasal fistula was technically feasible, we considered that such an approach would be inappropriate in this case, as it would not address the underlying self-injurious behavior and would likely result in recurrence of the palatal perforation. Therefore, we elected to use a soft palatal obturator made of ethylene-vinyl acetate, which is commonly employed for postoperative protection in patients with cleft palate. If an individually fabricated obturator could be accepted during meals, it was anticipated that food leakage into the nasal cavity could be prevented and that the patient's daily routine in the care facility could be maintained. The palatal obturator offers several advantages, including ease of insertion, removal, and maintenance, and functions as a plug-like device to close the perforation. In this case, the patient's diet mainly consisted of rice porridge, and the use of the obturator facilitated more comfortable oral intake. Both the patient and the care staff (caregivers and nurses) expressed satisfaction with its effectiveness during meals. In addition, the long-term increases in body weight and BMI, together with the absence of hospitalization due to aspiration pneumonia, suggest meaningful functional and nutritional improvement during the follow-up period. Furthermore, gradual reduction of crust formation around the nasal defect during the long-term follow-up suggests stabilization of the local condition and possible reduction of self-injurious behavior affecting the palate and nasal region. We speculate that the reduction in self-injurious behavior may be associated with decreased stress during eating, as prevention of food leakage allowed the patient to enjoy meals more comfortably, thereby contributing to psychological stabilization. The patient had no prior experience with dentures, and it was considered extremely difficult for her to accept complete dentures; therefore, prosthetic rehabilitation aimed at occlusal reconstruction was not attempted. Self-injurious behavior has been interpreted as a maladaptive coping mechanism to alleviate feelings of loneliness or emptiness, representing a distorted expression of the desire to live. The reasons why this patient engaged in self-injurious behavior specifically targeting the nasal region remain unclear. Medical interventions such as cryosurgery, electrocauterization for epistaxis, and nasal intubation are known potential causes of nasal septal perforation (2); however, none of these were present in this patient, making an iatrogenic cause unlikely. In 2015, a case of a rhesus macaque infected with cytomegalovirus developed neuropathy and exhibited self-injurious behavior targeting the nasal region (3). In humans, similar behavior involving the nasal region has been reported in patients with trigeminal trophic syndrome (4). These findings suggest that sensory dysfunction in the oral and maxillofacial region may contribute to self-injurious behavior involving the nose. However, in the present case, there was no history of neuropathy or trigeminal trophic syndrome, making these conditions unlikely as direct causes. Self-injurious behavior is widely observed in patients with psychiatric disorders, including factitious disorder (4), autism spectrum disorder (ASD) (5), and borderline personality disorder (6). Interestingly, sensory abnormalities, including olfactory dysfunction, have been reported in patients with ASD (7) as well as in ASD model mice (8). In 2018, when the patient was asked about her preference for curry, she responded positively to the question, "Do you like the taste of curry?" but showed no response to the question, "Do you like the smell of curry?" This observation may suggest a possible association between psychiatric dysfunction, altered sensory perception, and self-injurious behavior; however, the underlying mechanism remains unclear. The differential diagnosis of palatal perforation includes noma and syphilis. Noma is characterized by rapidly progressive gangrene and extensive tissue destruction (9), and cases have been reported in infants as young as 18 months of age (10). Numerous reports have also described palatal perforation associated with syphilis (11 , 12). In contrast, reports of self-inflicted palatal perforation are relatively rare, with five cases reported in Japan and several dozen cases identified in the PubMed database (Table 1) (4 , 13 - 30).

Table 1

Notably, only one case, reported in 1968, described the combined occurrence of nasal defect and palatal perforation due to self-injurious behavior (13). Although similar cases have previously been reported, the present case is clinically meaningful because of the exceptionally long-term follow-up and successful conservative management using a palatal obturator in a patient unsuitable for surgical treatment. Because the patient had intellectual disability, cognitive behavioral therapy for self-injurious behavior was considered difficult, and surgical closure of the palatal perforation was not performed. Instead, we selected a reversible and easily removable palatal obturator made of ethylene-vinyl acetate. The long-term use of the obturator contributed not only to maintenance of dietary quality of life, but also to improved nutritional status and stable general health during the approximately 18-year follow-up period.

## Figures and Tables

**Table 1:** Case reports of severe maxillofacial injuries caused by self-injurious behavior.

Reports from Japan					
Author (Year)			Findings		
Mizuno et al. (1986)			Rapid tongue enlargement due to tongue protrusion and self-biting		
Mori et al. (1990)			Extensive lower lip tissue loss and loss of mandibular primary incisors		
Morimoto et al. (1996)			Insertion of injection needle from mandible to cervical region		
Kawachi et al. (2022)			Self-inflicted injury with a razor resulted in a laceration of the tongue		
Saito et al. (2023)			Migration of piercing needle from tongue to submental subcutaneous tissue		
Present case (2026)			Oronasal fistula caused by repeated finger compression		
Reports searched from PubMed					
Year	Author	Title		Region	Ref. No.
1968	Gigliotti et al.	Self-inflicted destruction of nose and palate		Nose + Palate	13
1984	Fabiano et al.	Management of self-inflicted oral trauma		Tongue / Oral mucosa	14
1990	Croglio et al.	Self-inflicted oral trauma		Tongue / Lips	15
1992	Sheller	Self-inflicted oral trauma		Tongue	16
1994	Smith et al.	Lesch-Nyhan syndrome: case report		Tongue / Lips	17
1997	Rashid et al.	Oral self-mutilation in Lesch-Nyhan syndrome		Tongue	18
1999	Nurko et al.	Lip biting in Chiari II malformation		Lower lip	19
2000	Krejci	Self-inflicted gingival injury		Gingiva	20
2004	Tollefson et al.	Self-induced nasal ulceration		Nose	4
2004	Chevitarese et al.	Self-inflicted gingival injury		Gingiva	21
2004	Yasui et al.	Oral appliance for self-injury prevention		Tongue / Lips	22
2005	Cauwels et al.	Self-mutilation in Lesch-Nyhan syndrome		Tongue / Lips	23
2006	Jeong et al.	Preventive approach to oral self-mutilation		Tongue	24
2007	Guimarães et al.	Self-inflicted oral trauma in Moebius syndrome		Tongue / Lips	25
2010	Arhakis et al.	Treatment of self-injurious oral trauma		Tongue / Lips	26
2013	Limeres et al.	Oral self-injury: an update		Mixed oral sites	30
2014	Cannavale et al.	Oral self-injuries: series of 19 patients		Mixed	29
2018	González et al.	Oral self-mutilation in Lesch-Nyhan syndrome		Tongue / Lips	27
2022	Ferrão et al.	Oral self-mutilation: case report		Tongue / Lips	28

1
